# Clinical impact of computed tomography-measured skeletal muscle status at the third cervical vertebra on definitive radiotherapy outcomes for head and neck squamous cell carcinoma

**DOI:** 10.1007/s11604-025-01888-1

**Published:** 2025-10-03

**Authors:** Yuki Kasuga, Atsuto Katano, Masanari Minamitani, Shouhei Hanaoka, Shin Fujiwara, Yuki Saito, Koji Yamamura, Kenya Kobayashi, Hideomi Yamashita, Osamu Abe, Wataru Gonoi

**Affiliations:** 1 Department of Radiology, The University of Tokyo Hospital, 7-3-1 Hongo, Bunkyo-ku, Tokyo, 113-8655 Japan; 2 Department of Radiology, International University of Health and Welfare Narita Hospital, 4-2 Kozunomori, Narita-shi, Chiba, 286-8686 Japan; 3 Department of Otolaryngology—Head and Neck Surgery, The University of Tokyo Hospital, 7-3-1 Hongo, Bunkyo-ku, Tokyo, 113-8655 Japan

**Keywords:** Head and neck squamous cell carcinoma, Skeletal muscle area, Third cervical vertebra, Sarcopenia, Radiotherapy, Computed tomography

## Abstract

**Background and purpose:**

Loss of skeletal muscle mass is increasingly recognized as a poor prognostic indicator in patients with cancer, including those with head and neck squamous cell carcinoma (HNSCC). Emerging evidence suggests that the muscle area measured at the third cervical vertebra (C3) on CT serves as a practical surrogate for whole-body muscle status. This study aimed to evaluate the prognostic significance of C3-level body composition parameters in patients with HNSCC undergoing definitive radiotherapy.

**Materials and methods:**

A total of 283 consecutive patients with HNSCC treated with definitive radiotherapy between 2013 and 2023 were retrospectively analyzed. Pre-treatment CT scans were used to assess six body composition metrics at the C3 level: skeletal muscle area (SMA), mean skeletal muscle density, visceral adipose tissue area, subcutaneous adipose tissue area, visceral-to-subcutaneous fat ratio, and skeletal muscle fat infiltration index. Patients were stratified into high and low groups based on sex-specific median values. Associations between each body composition metric (high vs. low) and survival outcomes were assessed using univariate and multivariate analyses.

**Results:**

The cohort included 238 males and 45 females, with a median age of 67 years. Survival analysis showed a median follow-up period of 39.3 months. The 3-year overall survival (OS) rate for the entire cohort was 81.6%, and the 3-year progression free survival rate was 60.8%. In univariate analysis, only low SMA was significantly associated with poorer OS (hazard ratio: 1.841, *p* = 0.027). The median SMA was 35.0 cm^2^ for males and 23.5 cm^2^ for females. In multivariate analysis, low SMA remained an independent predictor of reduced OS (hazard ratio: 1.851, *p* = 0.028), along with clinical stage and chemotherapy status.

**Conclusion:**

Among the CT-derived body composition parameters assessed at the C3 level, low SMA was the only significant independent predictor of OS in patients with HNSCC receiving definitive radiotherapy. These findings support the clinical relevance of SMA assessment as a straightforward and robust prognostic biomarker and underscore the need for further investigation into the prognostic potential of other body composition metrics.

## Introduction

Head and neck cancer represents a significant global health burden, with data from the Global Burden of Disease database indicating a rising global incidence [1]. This increase appears more pronounced among females than males. Squamous cell carcinoma is the most prevalent histologic subtype of head and neck cancer, and treatment often necessitates a multimodal approach, including surgery, chemotherapy, and definitive radiotherapy. Despite advances in therapeutic strategies, treatment outcomes remain highly variable and are influenced by individual patient factors [2, 3]. Laboratory markers such as C-reactive protein and neutrophil-to-lymphocyte ratio have been identified as prognostic indicators in head and neck cancer [4–6]. Additionally, emerging research underscores the role of nutritional status and skeletal muscle mass in predicting treatment response and overall prognosis in patients with cancer [7–9].

Sarcopenia, defined by the progressive loss of skeletal muscle mass and function, has been increasingly recognized as a negative prognostic factor in various malignancies [10–12]. While sarcopenia is typically assessed using whole-body imaging, recent studies have emphasized the clinical utility of localized muscle assessments at specific anatomical landmarks. Measurements of skeletal muscle cross-sectional area (SMA) at the third lumbar (L3) vertebra, derived from clinical computed tomography (CT) scans, are commonly used for sarcopenia evaluation [13]. In patients with head and neck cancer, CT-based measurement of skeletal muscle at the third cervical vertebra (C3) has emerged as a reliable surrogate for whole-body muscle mass. Swartz et al. reported a strong correlation between muscle cross-sectional area at the C3 and L3 vertebral levels [14]. Their findings suggest that assessing skeletal muscle using head and neck CT scans is feasible and may serve as a practical alternative to abdominal imaging. This method offers a convenient and reproducible approach to evaluating muscle health in patients undergoing routine imaging for cancer diagnosis and treatment planning.

However, despite growing interest in C3-based assessments, their prognostic significance in patients with head and neck cancer receiving definitive radiotherapy remains incompletely understood. Moreover, the optimal cut-off values for defining sarcopenia using C3 skeletal muscle measurements are still debated. This study aims to evaluate the impact of CT-derived skeletal muscle status at the C3 vertebral level on outcomes following definitive radiotherapy in patients with head and neck squamous cell carcinoma (HNSCC). These insights may inform more individualized treatment approaches and improve outcomes for patients with HNSCC.

## Methods

### Patient selection

The present study included a consecutive series of 283 patients referred to our department for definitive radiotherapy for head and neck cancer between September 2013 and November 2023. The inclusion criteria were as follows: (1) histologically or cytologically confirmed squamous cell carcinoma; (2) primary tumors located in the hypopharynx, oropharynx, larynx, nasopharynx, paranasal sinuses, salivary glands, tongue, nasal cavity, or external auditory canal; (3) age over 18 years; (4) prescribed dose of 70 Gy in 35 fractions to the primary tumor and involved lymph nodes; (5) prophylactic lymph node regions treated using intensity-modulated radiotherapy (IMRT) with a simultaneously integrated boost; and (6) no prior curative-intent treatment for head and neck cancer. Clinical staging was based on the edition of the American Joint Committee on Cancer corresponding to the period of diagnosis. This study was approved by the Research Ethics Committee of The University of Tokyo Hospital (No: 3372-8) and adhered to the guidelines of the Strengthening the Reporting of Observational Studies in Epidemiology (STROBE) statement and the Declaration of Helsinki. We collected demographic and clinical data, including gender, age, Eastern Cooperative Oncology Group (ECOG) performance status (PS), clinical stage, and treatment regimens.

### Radiation therapy protocol

CT scans for radiation therapy planning were conducted while patients wore a thermoplastic mask for immobilization. The CT protocol involved a helical scan with a slice thickness of 2 mm at 120 kVp, and the tube current was 350 mA. Intravenous contrast medium was administered to 53 patients. The decision to use contrast was made at the discretion of the attending radiation oncologist, based on clinical context at the time of treatment planning. All patients received helical volumetric IMRT using TomoTherapy (Accuray Inc., Sunnyvale, CA, U.S.). Clinical target volume (CTV) 1 encompassed the primary tumor and any clinically positive lymph nodes [15]. Prophylactic irradiation of lymph node areas was guided by international recommendations [16]. CTV2 was defined as the ipsilateral elective nodal region, while CTV3 included the contralateral side. The planning target volume included the corresponding CTV with an additional 3–5 mm margin. Prescription doses using the Simultaneous Integrated Boost method were 70 Gy for PTV1, 59.5 Gy for PTV2, and 54 Gy for PTV3, all delivered in 35 fractions.

### CT image analysis of body composition parameters

Pre-treatment cross-sectional CT scans at the center of the C3 vertebral level were analyzed to assess skeletal muscle status [17]. The outlines of skeletal muscles in the selected images were manually traced to create regions of interest for muscle extraction. For contrast-enhanced CT scans, skeletal muscle area values were adjusted using the method proposed by Akamatsu et al., which enables estimation of non-contrast values from contrast-enhanced CT data to minimize measurement bias [18]. Within the skeletal muscle extraction regions of interest, pixels with attenuation values between −29 and 150 Hounsfield Units (HU) were extracted to quantify SMA (cm^2^), and the mean HU value was recorded as mean skeletal muscle density (MSMD, HU). Visceral adipose tissue area (VATA, cm^2^) was defined as the total area of adipose tissue with attenuation values from −150 to −50 HU, corresponding to visceral fat. Subcutaneous adipose tissue area (SATA, cm^2^) was similarly defined as adipose tissue with attenuation values between −190 and −30 HU, corresponding to subcutaneous fat. Both measurements were taken from the same axial CT slice. The visceral-to-subcutaneous fat area ratio (VSR) was calculated as VATA divided by SATA and was used as a surrogate marker for fat distribution. The skeletal muscle fat infiltration index was calculated as the proportion of pixels with attenuation values below −30 HU within the entire skeletal muscle region of interest. This value reflects the extent of intramuscular fat deposition, often referred to as “marbling” of the muscle.

### Data analysis

Statistical analyses were conducted using R software. Continuous variables were assessed using the Mann–Whitney *U* test. Categorical variables were compared using the chi-square test. Overall survival (OS) was defined as the duration from the start of radiotherapy to the date of death from any cause or the last follow-up. Progression free survival (PFS) was defined as the time during which a patient remained free of disease progression, with events including death or clinically or radiologically confirmed progression. Actuarial survival curves were generated using the Kaplan–Meier method and compared with the log-rank test. Multivariate analysis was performed to identify factors significantly associated with survival outcomes, with a *p* value < 0.05 indicating statistical significance.

## Results

In this single-institution study, 283 patients were included, comprising 238 males and 45 females. Patients’ characteristics are summarized in Table 1. The median age was 67 years (range: 18–89). ECOG PS was 0 in 223 patients and 1 in 60. Primary disease sites included hypopharyngeal cancer (*n* = 97), oropharyngeal cancer (*n* = 91), laryngeal cancer (*n* = 46), and nasopharyngeal cancer (*n* = 26). Approximately half of the cohort had early-stage disease (*n* = 134), with the remaining patients presenting advanced-stage disease (*n* = 149). p16 was positive in 63 patients and negative in 25, while 195 had unknown status because p16 testing is routinely done only for oropharyngeal cancer. Chemotherapy, either induction or concurrent, was administered to 186 patients.

**Table 1:** Summary of 283 patient characteristics in this present study

Variables	Number (percentage)
Age: median [range]	67 [18–89]
Gender	
Male	238 (84%)
Female	45 (16%)
ECOG performance status	
0	223 (79%)
1	60 (21%)
Primary disease	
Hypopharyngeal cancer	97 (34%)
Oropharyngeal cancer	91 (32%)
Laryngeal cancer	46 (16%)
Nasopharyngeal cancer	26 (9%)
Nasal and paranasal sinus cancer	15 (5%)
Others	8 (3%)
Clinical stage	
Early stage (I–II)	134 (47%)
Advanced stage (≥ III)	149 (53%)
Chemotherapy	
No	97 (34%)
Yes	186 (66%)

**Fig. 1:**
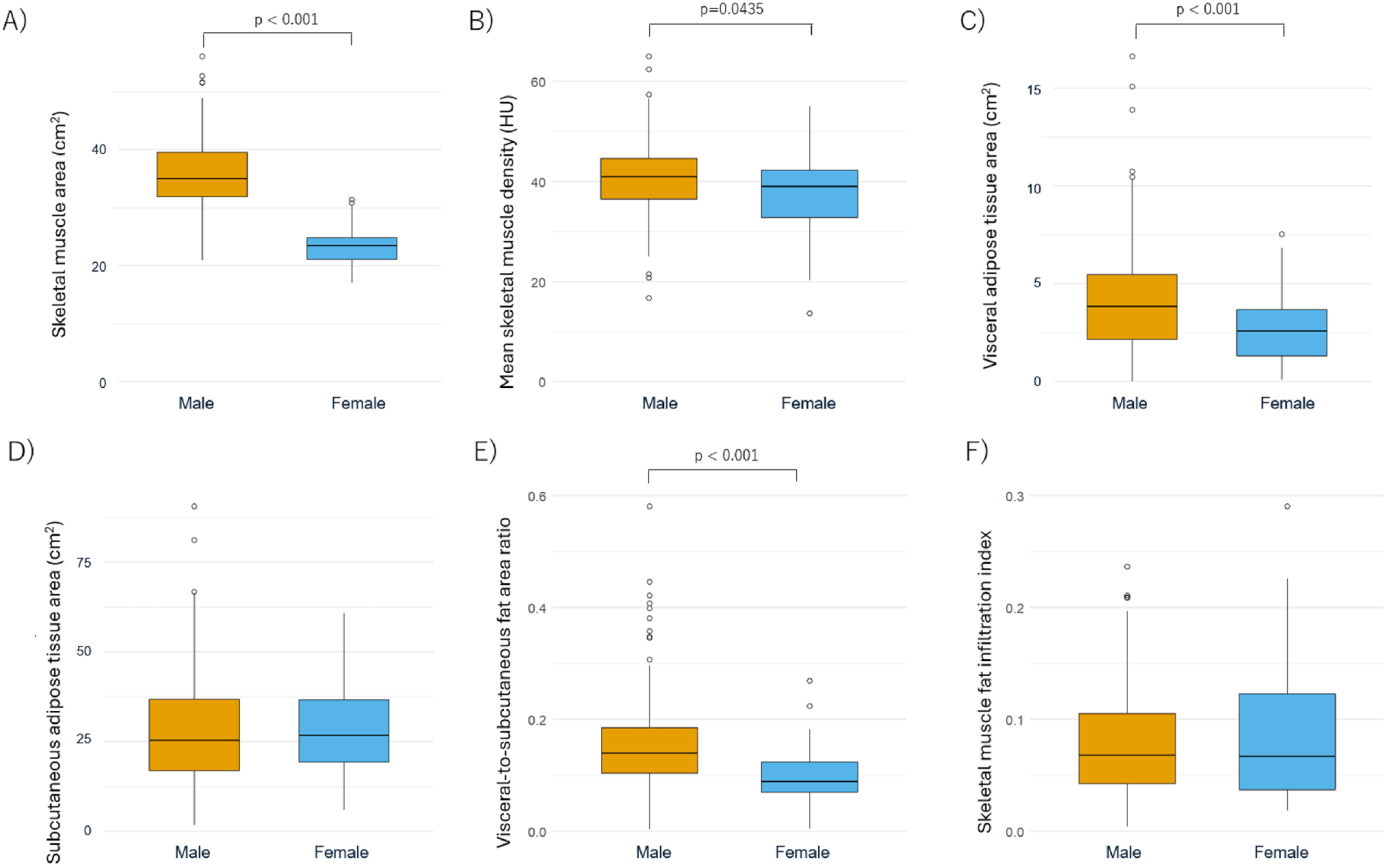
Sex-specific distribution of body composition parameters derived from CT imaging. The boxes represent the interquartile range (IQR), spanning from the first quartile (Q1) to the third quartile (Q3). Whiskers extend to 1.5 × IQR from the lower and upper quartiles. Data points lying outside this range were considered outliers and plotted individually as open circles. Comparison of body composition parameters between males and females: **A** skeletal muscle area, **B** mean skeletal muscle density, **C** visceral adipose tissue area, **D** subcutaneous adipose tissue area, **E** visceral-to-subcutaneous fat area ratio, and **F** skeletal muscle fat infiltration index

Among body composition parameters, median SMA was significantly higher in males (35.0 cm^2^) than females (23.5 cm^2^, *p* < 0.001), as were MSMD (41.0 vs. 39.0 HU, *p* = 0.0435), VATA (3.84 vs. 2.57 cm^2^, *p* < 0.001), and VSR (0.14 vs. 0.09, *p* < 0.001) [Fig. 1]. In contrast, no significant sex-based differences were observed for SATA (*p* = 0.634) or skeletal muscle fat infiltration index (p = 0.741). Patients were classified into high- and low-value groups for each parameter based on sex-specific median values; that is, an SMA > 35.0 cm^2^ for males and > 23.5 cm^2^ for females was defined as high-SMA, and values at or below these thresholds were defined as low-SMA. The same classification method was applied to MSMD, VATA, SATA, VSR, and skeletal muscle fat infiltration index.

**Fig. 2:**
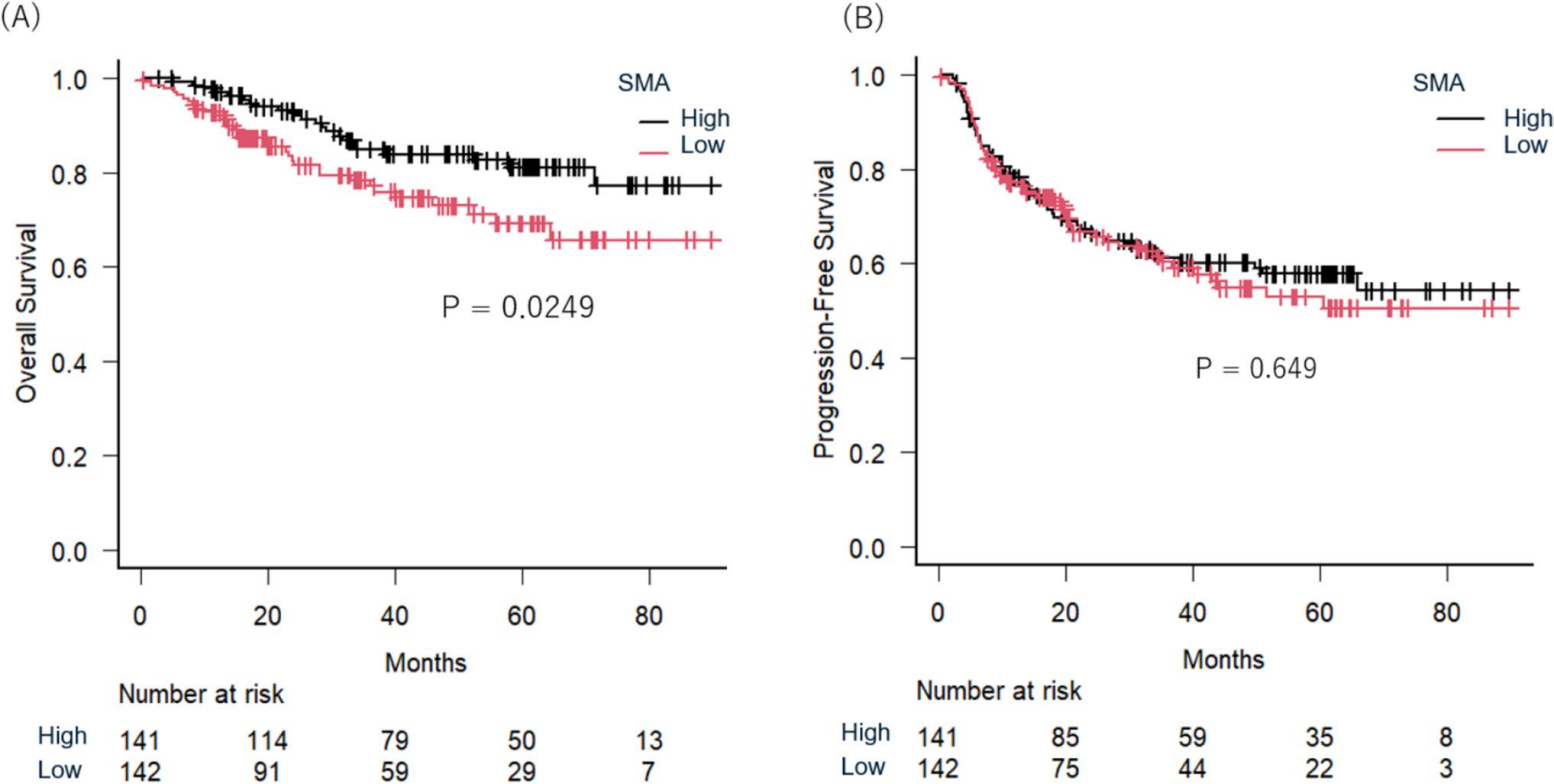
Kaplan–Meier survival plots stratified by skeletal muscle area (SMA) at the level of the third cervical vertebra, showing overall survival (**A**) and progression-free survival (**B**)

Median follow-up was 39.3 months (range: 0.2–112.4 months). Kaplan–Meier analysis showed the 3-year OS rate for the entire cohort was 81.6% (95% confidence interval (CI) 76.0–86.1%), and PFS was 60.8% (95% CI 54.4–66.5%). To investigate the prognostic significance of body composition parameters, univariable analyses were performed for OS and PFS [Table 2]. Among six parameters analyzed, only low SMA was significantly associated with poorer OS (hazard ratio (HR) = 1.841, 95% CI 1.071–3.162, *p* = 0.027) and was thus included in the multivariable model. Other body composition parameters showed no significant associations with OS or PFS. Log-rank analysis indicated that the low SMA group had significantly lower 3-year OS compared to the high SMA group (78.4% [95% CI 69.6–84.8%] vs. 84.9% [95% CI 77.1–90.3%], *p* = 0.0249) [Fig. 2A]. However, no significant difference in 3-year PFS between low and high SMA groups was observed (60.3% [95% CI 50.8–68.5%] vs. 61.1% [95% CI 52.1–69.0%], *p* = 0.649) [Fig. 2B].

**Table 2:** Univariate cox regression analysis of body composition parameters for overall survival and progression-free survival

Covariables	Value	Number	Overall survival		Progression free survival	
			Hazard ratio [95% CI]	*p* value	Hazard ratio [95% CI]	*p* value
Skeletal muscle area	High	141	1 (Reference)		1 (Reference)	
	Low	142	1.841 [1.071–3.162]	0.027	1.091 [0.751–1.583]	0.649
Mean skeletal muscle density	High	141	1 (Reference)		1 (Reference)	
	Low	142	1.272 [0.746–2.167]	0.377	1.174 [0.810–1.704]	0.397
Visceral adipose tissue area	High	141	1 (Reference)		1 (Reference)	
	Low	142	1.551 [0.904–2.661]	0.111	1.142 [0.788–1.655]	0.484
Subcutaneous adipose tissue area	High	141	1 (Reference)		1 (Reference)	
	Low	142	1.325 [0.777–2.257]	0.301	1.130 [0.780–1.637]	0.519
Visceral-to-subcutaneous fat area ratio	High	141	1 (Reference)		1 (Reference)	
	Low	142	1.022 [0.602–1.735]	0.936	1.001 [0.690–1.453]	0.995
Skeletal muscle fat infiltration index	High	141	1 (Reference)		1 (Reference)	
	Low	142	1.025 [0.604–1.740]	0.927	1.155 [0.796–1.677]	0.449

In addition to SMA, poor PS (HR = 2.263, 95% CI 1.287–3.981, *p* = 0.005) and advanced clinical stage (HR = 3.716, 95% CI 1.993–6.927, *p* < 0.001) were significant predictors of OS in univariable analysis [Table 2]. Multivariate analysis confirmed low SMA as an independent predictor of poorer OS (HR = 1.851, 95% CI 1.070–3.200, *p* = 0.028), along with clinical stage and receipt of chemotherapy. For PFS, multivariate analysis identified PS, clinical stage, and chemotherapy administration as independent prognostic factors, while SMA did not reach statistical significance (HR = 1.070, 95% CI 0.736–1.556, *p* = 0.722) [Table 3].

**Table 3:** Univariate and multivariate Cox proportional hazards analysis of overall survival

Covariables	Value	Number	Univariate		Multivariate	
			Hazard ratio [95% CI]	*p* value	Hazard ratio [95% CI]	*p* value
Age	(Continuous variable)		1.017 [0.989–1.046]	0.233	0.995 [0.966–1.025]	0.748
Gender	Male	238	1 (Reference)		1 (Reference)	
	Female	45	1.282 [0.646–2.547]	0.478	1.339 [0.653–2.746]	0.426
Performance status	0	223	1 (Reference)		1 (Reference)	
	1	60	2.263 [1.287–3.981]	0.005	1.396 [0.774–2.518]	0.267
Clinical stage	I–II	134	1 (Reference)		1 (Reference)	
	≥ III	149	4.721 [2.378–9.373]	< 0.001	9.297 [4.014–21.530]	< 0.001
Chemotherapy	No	97	1 (Reference)		1 (Reference)	
	Yes	186	0.940 [0.539–1.639]	0.828	0.264 [0.124–0.565]	0.001
SMA	High	141	1 (Reference)		1 (Reference)	
	Low	142	1.841 [1.071–3.162]	0.027	1.851 [1.070–3.200]	0.028

*SMA* skeletal muscle area at the level of the third cervical vertebra

**Fig. 3:**
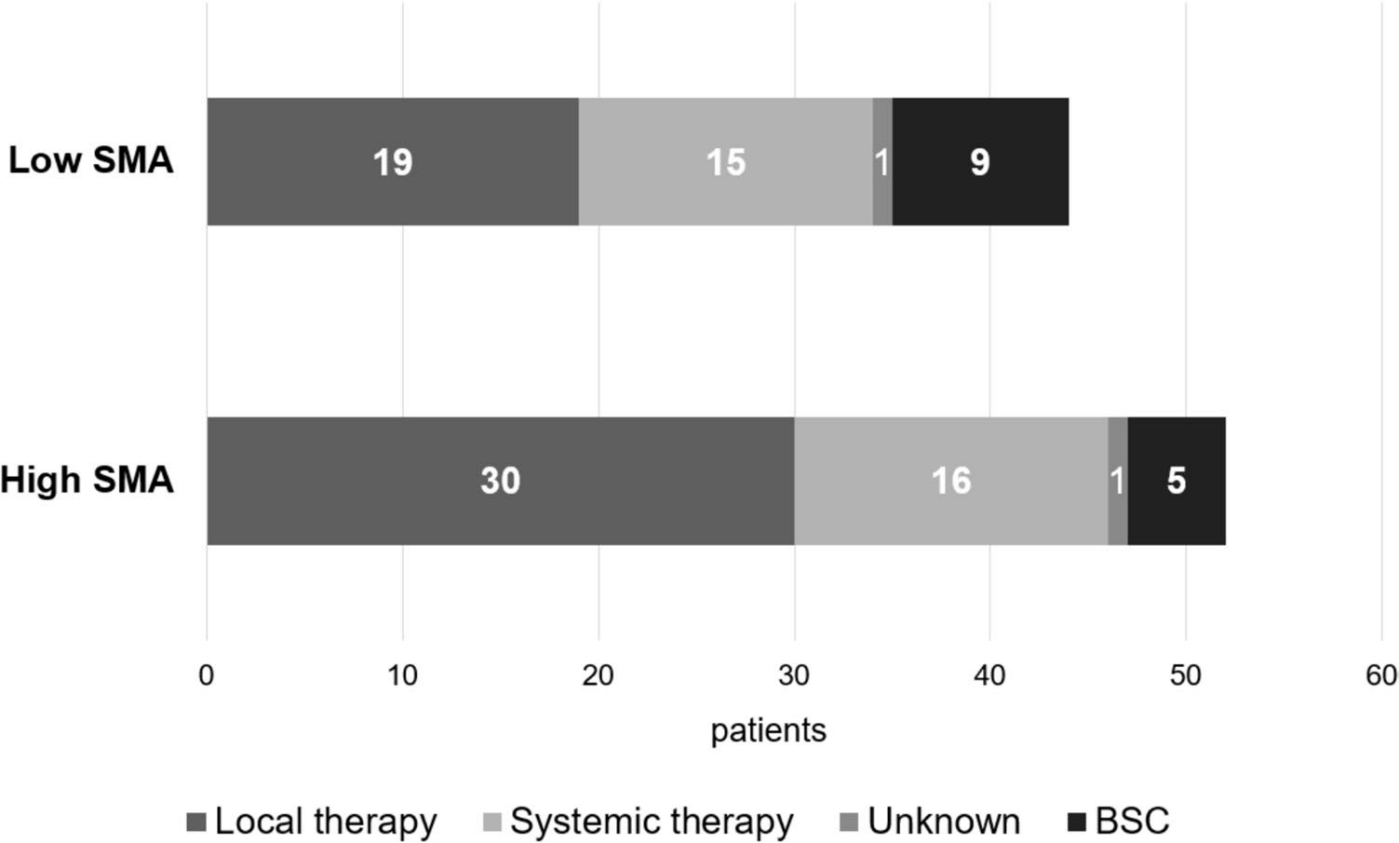
Salvage therapy after disease progression in low skeletal muscle area (SMA) group and high SMA group

Disease progression occurred in 96 patients: 44 in the low SMA group and 52 in the high SMA group. Salvage treatments are summarized in Fig. 3. Local therapies, including surgery, stereotactic radiotherapy, and photoimmunotherapy, were more frequently administered in the high SMA group (30 of 52 patients, 57.7%) compared to the low SMA group (19 of 44 patients, 43.1%) (*p* = 0.137). Systemic therapy was slightly more common in the low SMA group (15 patients, 34%) than in the high SMA group (16 patients, 29.1%) (*p* = 0.664). Best supportive care (BSC) was utilized more frequently in the low SMA group (9 patients, 20.4%) compared to the high SMA group (5 patients, 9.6%) (*p* = 0.138) (See Table 4).

**Table 4:** Univariate and multivariate Cox proportional hazards analysis of progression free survival

Covariables	Value	Number	Univariate		Multivariate	
			Hazard ratio [95% CI]	*p* value	Hazard ratio [95% CI]	*p* value
Age	(Continuous variable)		0.993 [0.976–1.011]	0.463	0.985 [0.967–1.004]	0.118
Gender	Male	238	1 (Reference)		1 (Reference)	
	Female	45	1.054 [0.636–1.747]	0.840	0.968 [0.579–1.618]	0.900
Performance status	0	223	1 (Reference)		1 (Reference)	
	1	60	1.966 [1.302–2.969]	0.001	1.563 [1.021–2.395]	0.040
Clinical stage	I–II	134	1 (Reference)		1 (Reference)	
	≥ III	149	2.405 [1.616–3.577]	< 0.001	3.317 [1.996–5.512]	< 0.001
Chemotherapy	No	97	1 (Reference)		1 (Reference)	
	Yes	186	1.037 [0.701–1.533]	0.857	0.454 [0.269–0.768]	0.003
SMA	High	141	1 (Reference)		1 (Reference)	
	Low	142	1.091 [0.751–1.583]	0.649	1.070 [0.736–1.556]	0.722

*SMA* skeletal muscle area at the level of the third cervical vertebra

## Discussion

The present study elucidated the prognostic significance of skeletal muscle status, as assessed by CT at the C3 vertebral level, in patients with HNSCC undergoing definitive radiotherapy. Our findings demonstrate that low SMA is significantly associated with poorer OS, reinforcing the role of muscle mass as a clinically meaningful prognostic marker. While low SMA predicted reduced OS, no significant difference in PFS was observed between the high- and low-SMA groups. Furthermore, analysis of disease progression revealed that patients with low SMA were more likely to receive best supportive care after recurrence, suggesting that diminished muscle mass may influence subsequent treatment decisions. In interpreting our findings, it is important to note the rationale for dichotomizing body composition parameters. The distributions of C3-derived body composition metrics differed substantially between men and women, as shown in Fig. 1. Therefore, modeling these parameters as a single continuous variable would not be appropriate. We therefore employed a sex-specific median split as the primary analytic strategy.

Multivariate analysis confirmed low SMA as an independent predictor of OS, alongside clinical stage and receipt of chemotherapy. For PFS, however, PS, clinical stage, and chemotherapy were independent prognostic factors, while SMA did not reach statistical significance. Kise et al. evaluated sarcopenia in 134 women with cervical squamous cell carcinoma undergoing chemoradiotherapy, using the psoas muscle index (cross-sectional psoas muscle area normalized to height squared) as a prognostic indicator [19]. They similarly reported that psoas muscle index independently predicted OS but not PFS, aligning with our findings. In our cohort, despite the absence of statistical significance (*p* = 0.137), local salvage therapies were more frequent in the high-SMA group (57.7% vs. 43.1%), suggesting that higher muscle mass may enhance tolerance for aggressive interventions, potentially improving OS.

Several studies consistently support our findings that low muscle mass negatively impacts OS in patients with head and neck cancer. Deantoni et al. investigated the prognostic impact of skeletal muscle mass index (SMI), which was defined as SMA divided by height squared, at C3 in 76 patients with advanced oropharyngeal squamous cell carcinoma treated with Helical TomoTherapy [20]. They found that OS was significantly lower in patients with low SMI, particularly in those with less advanced nodal disease. Haehl et al. examined the impact of sarcopenia on elderly patients with HNSCC undergoing chemoradiation, defining sarcopenia based on the muscle area manually outlined and quantified on CT scans at the level of the C3 in a cohort of 280 patients [21]. In their cohort, sarcopenic patients had significantly reduced OS (23 vs. 91 months), and sarcopenia remained an independent predictor of poor OS. Compared with previous studies, our study offers several notable strengths. First, we analyzed a relatively large cohort of 283 patients who were uniformly treated with a standardized definitive radiotherapy protocol, thereby minimizing treatment-related variability. Second, we comprehensively evaluated multiple CT-derived body composition parameters and demonstrated that only SMA was independently associated with overall survival. Third, we assessed post-recurrence treatment patterns and found that patients with higher SMA were more likely to receive salvage therapy, suggesting that skeletal muscle status may influence not only prognosis but also therapeutic decisions following disease progression.

Although our study did not reach statistical significance regarding PFS, patients in the low SMA group showed a trend toward poorer outcomes, likely attributable to insufficient statistical power. Rijn-Dekker et al. reported significant associations between sarcopenia and reduced OS (HR 0.72, *p* = 0.012) and disease-free survival (DFS) (HR 0.67, *p* = 0.001) among 750 patients treated with definitive radiotherapy for HNSCC [22]. Furthermore, Takenaka et al. conducted a meta-analysis of 18 studies #involving 3,233 patients, confirming sarcopenia’s prognostic relevance across various treatment modalities, including surgery and radiation [23]. Unlike previous meta-analyses, our study specifically examined patients undergoing definitive radiotherapy, utilizing skeletal muscle measurements at the C3 level from planning CT scans, and explored treatment patterns post-recurrence, providing novel insights into how muscle status influences therapeutic decisions.

Some prior studies have proposed C3-based approaches to approximate L3 muscle metrics. Swartz et al. derived a regression model that estimates L3 skeletal muscle cross-sectional area from the C3 area with age, weight, and sex as covariates [14]. Olson et al. reported sex-specific C3 skeletal muscle index cut-offs that best predicted L3-defined sarcopenia in their cohort [17]. However, the transportability of these standards to Asian populations is uncertain. In a Korean cohort, Yoon et al. found a weak correlation between C3 and L3 skeletal muscle measures in sarcopenic patients and a prediction model with poor diagnostic accuracy [24]. Alignment with these published standards and external validation in large, multi-institutional cohorts will be essential.

Certain limitations should be acknowledged. First, this was a retrospective, single-center study, which may introduce selection and information bias and limits generalizability. Second, the cohort was heterogeneous across primary head and neck subsites, particularly due to inclusion of nasopharyngeal carcinoma and nasal/paranasal carcinomas, reducing comparability with more homogeneous trial populations [25–27]. Third, p16 status was available for only about 30% of patients, precluding p16-related analyses such as covariate adjustment and stratification. Fourth, using skeletal muscle measurements at the C3 vertebral level introduces variability related to patient positioning during imaging [28]. Evaluating sarcopenia radiologically is further complicated by the absence of standardized measurement protocols and diagnostic cutoff points [29, 30]. As prognostic factors derived from our retrospective analyses requires validation before clinical application, future studies with internal or external validation are warranted. In particular, external validation using larger, multi-institutional cohorts is desirable to confirm the reproducibility and generalizability.

In conclusion, this study underscores the clinical importance of assessing skeletal muscle status at the C3 level in patients with head and neck cancer undergoing definitive radiotherapy. This approach offers a practical and less burdensome alternative to whole-body imaging and yields clinically valuable insights for individualized therapeutic strategies. Integrating muscle assessments into routine clinical practice may improve management and outcomes, especially for patients experiencing sarcopenia. Future studies should further clarify the role of skeletal muscle in predicting treatment response and prognosis, refining therapeutic strategies for this complex patient population.
